# Activity Recognition Invariant to Sensor Orientation with Wearable Motion Sensors

**DOI:** 10.3390/s17081838

**Published:** 2017-08-09

**Authors:** Aras Yurtman, Billur Barshan

**Affiliations:** Department of Electrical and Electronics Engineering, Bilkent University, Bilkent, Ankara 06800, Turkey; yurtman@ee.bilkent.edu.tr

**Keywords:** human activity recognition, wearable sensing, sensor orientation, orientation-invariant sensing, motion sensors, inertial sensors, accelerometer, gyroscope, magnetometer, singular value decomposition, machine learning, Bayesian decision making, k-nearest-neighbor classifier, support vector machines, artificial neural networks

## Abstract

Most activity recognition studies that employ wearable sensors assume that the sensors are attached at pre-determined positions and orientations that do not change over time. Since this is not the case in practice, it is of interest to develop wearable systems that operate invariantly to sensor position and orientation. We focus on invariance to sensor orientation and develop two alternative transformations to remove the effect of absolute sensor orientation from the raw sensor data. We test the proposed methodology in activity recognition with four state-of-the-art classifiers using five publicly available datasets containing various types of human activities acquired by different sensor configurations. While the ordinary activity recognition system cannot handle incorrectly oriented sensors, the proposed transformations allow the sensors to be worn at any orientation at a given position on the body, and achieve nearly the same activity recognition performance as the ordinary system for which the sensor units are not rotatable. The proposed techniques can be applied to existing wearable systems without much effort, by simply transforming the time-domain sensor data at the pre-processing stage.

## 1. Introduction

Human activity recognition has been an active field of research since the late 1990s, with applications in medical, surveillance, entertainment, and military systems [1,2,3]. Because of the increasing capabilities of mobile devices and wireless sensors, using wearables in activity recognition has gained considerable advantages over external sensors (e.g., cameras, vibration, and acoustic sensors) placed in a restricted environment [4]. Earlier works on activity recognition that employ wearable sensors are reviewed in [5,6,7]. In most applications of wearable sensing, it is assumed that sensors are placed at pre-determined positions and orientations that remain fixed over time [8]. This assumption may be obtrusive because the user needs to be attentive to placing the sensor and to keeping it at the same position and orientation. In practice, users may place the sensors incorrectly on the body and their positions and orientations can gradually change because of loose attachments and body movement. If the sensors are worn on specially designed clothing or accessories, these may vibrate or move relative to the body. Often, elderly, disabled, or injured people also need to wear these sensors [9], and may have difficulty positioning them or adjusting the position once attached. Hence, position- and orientation-invariant sensors would be advantageous for these types of users as well.

Incorrect placement of a wearable sensor includes both placing it at a different position and at a different orientation. It would be a valuable contribution to develop wearable systems that are invariant to sensor position and orientation without a significant degradation in performance. In the former, sensor units can be placed anywhere on the same body part (e.g., lower arm) or on different body parts; in the latter, the units can be fixed to pre-determined positions at any orientation. Studies exist that consider both position and orientation invariance at the same time but none of these works can handle incorrect placement of sensors without a considerable loss in performance (between 20–50%) [10]. Thus, these two problems have not been solved completely in existing studies. In particular, the studies on orientation-invariant sensing have strong limitations and have been tested in very restricted scenarios. Hence, we focused on the latter problem and developed two different *orientation-invariant transformations* (OITs) for the generic activity recognition scheme that can be easily adapted to existing systems. Assuming the sensors are placed at the correct positions on the body to begin with, they can function in an orientation-invariant manner without significant accuracy reduction. Our approaches make minimal assumptions about sensor types and make no assumptions about the usage scenario and the sensor configuration. The techniques are based on transforming 3-D time-domain sequences to other multi-D time-domain sequences that are invariant to absolute sensor orientation. In this way, the transformed data become independent of the absolute orientations of the multi-axial sensors, allowing subjects to fix the sensor units on their bodies at any orientation. Using the transformed data, any algorithm that can be applied to multi-D time-domain sequences can be used in an orientation-invariant manner. We tested the proposed approach on activity recognition with five publicly available datasets, one of which was acquired by our research group [11]. We observed that the degradation in activity recognition accuracy is acceptable for both OIT techniques, even though the absolute orientation information of the sensor unit is not preserved in the transformation.

The rest of this article is organized as follows: In Section 2, we summarize the related work on orientation-invariant sensing. We describe the proposed transformations in Section 3. In Section 4, we briefly introduce the datasets and present the experimental results comparatively. We discuss the proposed methodology and compare it with the existing studies in Section 5. We draw conclusions and provide directions for future research in Section 6.

## 2. Related Work

Despite numerous studies on activity recognition with wearables, there are relatively few studies that focus on wearable sensing invariant to sensor orientation [3,12]. Some studies estimate the vertical and forward-backward (saggital) axes of the body so that the acquired sensor data can be rotated and expressed in the body coordinate frame. This method requires the sensors to be located on the chest or the waist because other parts of the body, such as the head or the limbs, often rotate irregularly with respect to the trunk. References [13,14] estimate the direction of the body’s vertical axis by averaging the 3-D acceleration sequence based on the assumption that the long-term mean of the acceleration should be along the direction of the gravity vector during daily activities. Thus, in [13], the acceleration component along the gravity vector and the magnitude of the acceleration on the plane perpendicular to that are used for classification. Reference [14] estimates the direction of the forward-backward axis as the principal axis of the data projected onto the plane perpendicular to the vertical axis, assuming that most of the body movements are along the aforementioned axis. Hence, it uses 3-D rotated sensor data in the body coordinate frame. Both studies consider six daily activities and follow the standard activity recognition paradigm, consisting of segmentation, feature extraction, and classification [15]. In [13], accuracy values up to 90% are achieved, but it is also shown that similar accuracy values can be obtained by simply using the magnitude (Euclidean norm) of the acceleration vector, which is invariant to sensor orientation. In [14], 16 different sensor orientations are considered, assuming that a mobile phone equipped with an accelerometer can be placed into a pocket only at these orientations. The proposed rotation from the sensor to the body frame increases the classification accuracy from 80.6% to 86.4%. Reference [16] obtains the axes in the transformed coordinate frame by applying principal component analysis (PCA) to the acceleration and gyroscope sequences. High accuracy is obtained in a small dataset acquired from a smartphone that contains five activities and 125 time segments in total.

In [17,18,19,20,21,22], sensor orientation invariance in activity recognition is achieved by using the magnitude of the tri-axial acceleration vector. The former study [17] uses support vector machines (SVM) to classify the features extracted from the time segments. For six sensor positions, each with four orientations, the proposed approach slightly increases the precision, recall, and *F*-score by about 1.5% compared to using the acceleration vector only, which is also quite accurate. Possible reasons for the small improvement are the robustness of the SVM classifier, the limited number of orientations, and the large size of the dataset. Reference [23] considers four different orientations for a tri-axial accelerometer on the waist, with the *x* axis always pointing in the same direction. The dynamic portions of the sequences are extracted and sensor orientation is recognized by a one-nearest-neighbor (1-NN) classifier with 100% accuracy. Then, the sensor data are rotated accordingly and activities are recognized. However, orientation classification may not work accurately if more sensor orientations are included or if the user performs activities that are not considered.

Reference [24] transforms the acquired data from the sensor coordinate frame to the Earth’s coordinate frame by estimating the sensor orientation for each time sample because data represented in the Earth frame are invariant to the sensor orientation. The sensor orientation is estimated based on the gyroscope sequences in the short term and from the directions of gravity and the Earth’s magnetic field measured by the accelerometer and the magnetometer, respectively, in the long term. Hence, this technique requires accelerometer, gyroscope, and magnetometer sensors. Although this method is shown to obtain an accuracy close to the fixed sensor orientation case, only two different sensor orientations are considered.

Reference [10] considers the case where some of the sensor units are placed incorrectly on the same body part and proposes an activity recognition method that may tolerate this. Two types of incorrect placement are considered: fixing the sensor at any orientation at the correct position by the user, and at any orientation and position on the same body part (e.g., lower leg). Instead of aggregating features obtained from multiple sensor units, an individual classifier for each unit is trained and the decisions are fused. Then, incorrect placement of a sensor affects the decision of only one classifier, but it will not be confident about its (possibly incorrect) decision; hence, the final decision will rarely be affected. It is observed that the best-performing technique for a problem with 30 activities is using the *k*-nearest-neighbor (*k*-NN) classifier on the most complicated feature set, but the accuracy decreases by more than 25% when seven of the nine sensor units are placed incorrectly on the same body part (both incorrect orientation and position). For a single-sensor setup, incorrect orientation of the sensor decreases the accuracy by 20–25% [10], whereas the accuracy is lower than 50% in all the scenarios for both incorrect sensor orientation and positioning on the same body part. Therefore, incorrect placement of even some of the sensors still causes a considerable drop in accuracy, and this cannot be tolerated in a single-sensor scenario.

In some studies that consider position invariance in wearable sensing [25,26], it is assumed that a sensor can be placed at different positions on the same body part but the orientation is assumed to remain the same, which is not realistic. In [25], activities are recognized independent of sensor positions but the deviations or ambiguities in sensor orientation are completely neglected, claiming that “in most cases, [sensor] orientation can be estimated with reasonable effort” [25]. This claim has not been proven to be true in the existing studies on wearable sensing that consider orientation invariance and could only have been achieved in restricted scenarios with strong assumptions.

Another related work [27] proposes a transformation on 2-D trajectories to achieve rotational invariance. The transformation yields a single non-uniformly sampled 1-D sequence which needs to be interpolated and re-sampled. The method is applied to a handwriting dataset recorded with a tablet where the letters are considered to be 2-D trajectories. This method is not directly applicable in 3-D where the sensor units can freely rotate about three orthogonal axes, unlike the simpler 2-D case where the rotation is about a single axis.

To summarize, existing approaches are aimed at specific scenarios and cannot be applied to a generic activity recognition scheme. These works make strong assumptions about the possible sensor orientations, sensor types, and the nature of the activities, which are not applicable to most activity recognition problems.

## 3. Invariance to Sensor Orientation

To achieve orientation-invariant activity recognition with acceptable accuracy, we propose to transform the 3-D time-domain sensor data in a way that the resulting sequences do not depend on absolute sensor orientation (but they *should* depend on the *changes* in the orientation over time to preserve activity-related rotational information). In other words, each 3-D time-domain sensor sequence is transformed to another multi-D time-domain sequence in an orientation-invariant manner.

We propose two different OIT techniques, namely the *heuristic* OIT and the *singular value decomposition (SVD)-based* OIT, described below.

### 3.1. Heuristic Orientation-Invariant Transformation

In the heuristic OIT, 3-D sensor data are transformed into 9-D data, invariant to sensor orientation. Let ${\vec{v}}_{n}={\left(v_{x}\left[n\right],v_{y}\left[n\right],v_{z}\left[n\right]\right)}^{T},\;\;\;0\leq n\leq N$ be the data vector in 3-D space $R^{3}$ acquired from the x,y,z axes of a tri-axial sensor, such as an accelerometer, at time sample *n*. The first- and second-order time-differences of ${\vec{v}}_{n}$ are defined as $\Delta {\vec{v}}_{n}={\vec{v}}_{n+1}-{\vec{v}}_{n}$ and $\;\Delta \;\;\;\;\;\;\Delta \;{\vec{v}}_{n}=\Delta {\vec{v}}_{n+1}-\Delta {\vec{v}}_{n}$, respectively. The heuristic OIT, represented by a transformation $T_{\mathrm{heuristic}}:{\vec{v}}_{n}\to {\vec{w}}_{n}\;\;\;\;\forall n$, transforms the measurement vectors ${\vec{v}}_{n}\in R^{3}$ to orientation-invariant vectors ${\vec{w}}_{n}\in R^{9}$, whose elements are selected as follows:

(1a) $$\begin{array}{ccc} w_{1}\left[n\right] & =∥{\vec{v}}_{n}∥ & \left(\mathrm{the}\;\mathrm{norm}\right) \end{array}$$

(1b) $$\begin{array}{ccc} w_{2}\left[n\right] & =∥\Delta {\vec{v}}_{n}∥ & \left(\mathrm{the}\;\mathrm{norm}\;\mathrm{of}\;\mathrm{the}\;\mathrm{first-order}\;\mathrm{difference}\;\Delta {\vec{v}}_{n}\right) \end{array}$$

(1c) $$\begin{array}{ccc} w_{3}\left[n\right] & =∥\;\Delta \;\;\;\;\;\;\Delta \;{\vec{v}}_{n}∥ & \left(\mathrm{the}\;\mathrm{norm}\;\mathrm{of}\;\mathrm{the}\;\mathrm{second-order}\;\mathrm{difference}\;\;\Delta \;\;\;\;\;\;\Delta \;{\vec{v}}_{n}\right) \end{array}$$

(1d) $$\begin{array}{ccc} w_{4}\left[n\right] & =\alpha_{n}=∠\left({\vec{v}}_{n},{\vec{v}}_{n+1}\right) & \left(\mathrm{the}\;\mathrm{angle}\;\mathrm{between}\;{\vec{v}}_{n}\;\mathrm{and}\;{\vec{v}}_{n+1}\right) \end{array}$$

(1e) $$\begin{array}{ccc} w_{5}\left[n\right] & =\beta_{n}=∠\left(\Delta {\vec{v}}_{n},\Delta {\vec{v}}_{n+1}\right) & \left(\mathrm{the}\;\mathrm{angle}\;\mathrm{between}\;\Delta {\vec{v}}_{n}\;\mathrm{and}\;\Delta {\vec{v}}_{n+1}\right) \end{array}$$

(1f) $$\begin{array}{ccc} w_{6}\left[n\right] & =\gamma_{n}=∠\left(\;\Delta \;\;\;\;\;\;\Delta \;{\vec{v}}_{n},\;\Delta \;\;\;\;\;\;\Delta \;{\vec{v}}_{n+1}\right) & \left(\mathrm{the}\;\mathrm{angle}\;\mathrm{between}\;\;\Delta \;\;\;\;\;\;\Delta \;{\vec{v}}_{n}\;\mathrm{and}\;\;\Delta \;\;\;\;\;\;\Delta \;{\vec{v}}_{n+1}\right) \end{array}$$

(1g) $$\begin{array}{ccc} w_{7}\left[n\right] & =\theta_{n}=∠\left({\vec{p}}_{n},{\vec{p}}_{n+1}\right)\;\mathrm{where}\;{\vec{p}}_{n}={\vec{v}}_{n}\times {\vec{v}}_{n+1} & \left(\mathrm{the}\;\mathrm{angle}\;\mathrm{between}\;\mathrm{rotation}\;\mathrm{axes}\;{\vec{p}}_{n}\;\mathrm{and}\;{\vec{p}}_{n+1}\right) \end{array}$$

(1h) $$\begin{array}{ccc} w_{8}\left[n\right] & =\phi_{n}=∠\left({\vec{q}}_{n},{\vec{q}}_{n+1}\right)\;\mathrm{where}\;{\vec{q}}_{n}=\Delta {\vec{v}}_{n}\times \Delta {\vec{v}}_{n+1} & \left(\mathrm{the}\;\mathrm{angle}\;\mathrm{between}\;\mathrm{rotation}\;\mathrm{axes}\;{\vec{q}}_{n}\;\mathrm{and}\;{\vec{q}}_{n+1}\right) \end{array}$$

(1i) $$\begin{array}{ccc} w_{9}\left[n\right] & =\psi_{n}=∠\left({\vec{r}}_{n},{\vec{r}}_{n+1}\right)\;\mathrm{where}\;{\vec{r}}_{n}=\;\Delta \;\;\;\;\;\;\Delta \;{\vec{v}}_{n}\times \;\Delta \;\;\;\;\;\;\Delta \;{\vec{v}}_{n+1} & \left(\mathrm{the}\;\mathrm{angle}\;\mathrm{between}\;\mathrm{rotation}\;\mathrm{axes}\;{\vec{r}}_{n}\;\mathrm{and}\;{\vec{r}}_{n+1}\right) \end{array}$$

The rationale for selecting these nine elements among many is that apart from the norms covered by the first three elements, the angles between the successive time samples of the sensor sequence and its first- and second-order differences (fourth–sixth elements) contain more granularity and fine detail regarding the activities performed. The last three elements consider rotation axes between successive time samples and contain information about the rotational movements of the data vectors in 3-D space.

**Figure 1:**
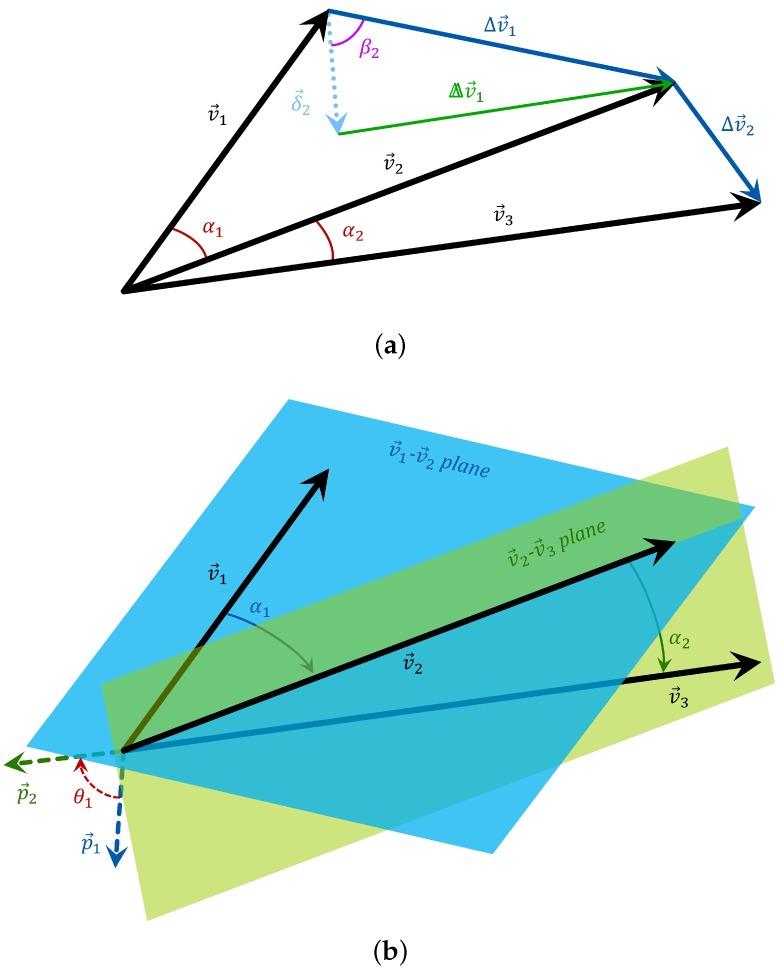
Graphical illustration of the selected axes of the heuristic orientation-invariant transformation (OIT). The geometric features of three sequential measurements ${\vec{v}}_{1},{\vec{v}}_{2},{\vec{v}}_{3}$ in 3-D space are shown. The first- and second-order difference sequences, the angles between successive measurement vectors, and the angles between successive difference vectors are shown in (**a**); The rotation axes and the angles between them are illustrated in (**b**).

The first five elements are shown geometrically in Figure 1a. In Equation (1) and throughout this text, $∥\cdot ∥$ denotes the Euclidean norm. In Equation (1d), the angle $\alpha_{n}$ between ${\vec{v}}_{n}$ and ${\vec{v}}_{n+1}$ is calculated based on the two vectors’ normalized inner product:

(2) $$\alpha_{n}=∠\left({\vec{v}}_{n},{\vec{v}}_{n+1}\right)=\cos^{-1}\left(\frac{{\vec{v}}_{n}\cdot {\vec{v}}_{n+1}}{∥{\vec{v}}_{n}∥∥{\vec{v}}_{n+1}∥}\right)$$

The angle $\alpha_{n}$ is set to zero when ${\vec{v}}_{n}=\vec{0}$ and/or ${\vec{v}}_{n+1}=\vec{0}$, in which case it is not defined. The angles in Equation (1e–i) are calculated in the same way.

In Equation (1g), ${\vec{p}}_{n}$ is the vector representing the axis of rotation from ${\vec{v}}_{n}$ to ${\vec{v}}_{n+1}$; that is, ${\vec{v}}_{n+1}$ is obtained when ${\vec{v}}_{n}$ is rotated about ${\vec{p}}_{n}$ by an angle of $\alpha_{n}$ (see Equation (1d) and Figure 1b). Similarly, ${\vec{v}}_{n+2}$ is obtained when ${\vec{v}}_{n+1}$ is rotated about ${\vec{p}}_{n+1}$ by $\alpha_{n+1}$. Then, the angle between the consecutive rotation axes, ${\vec{p}}_{n}$ and ${\vec{p}}_{n+1}$, is calculated, which is denoted by $\theta_{n}$, as shown in Figure 1b. In Equation (1h,i), the rotation axes are calculated based on the first- and second-order difference sequences $\Delta {\vec{v}}_{n}$ and $\;\Delta \;\;\;\;\;\;\Delta \;{\vec{v}}_{n}$, respectively, and the angle between the consecutive rotation axes is calculated. (${\vec{p}}_{n},{\vec{q}}_{n}$, and ${\vec{r}}_{n}$ need not have unit norms because only their directions are used in Equation (1g–i).)

The transformed vector ${\vec{w}}_{n}$ has nine elements, corresponding to the new axes that are completely invariant to sensor orientation. Mathematically, when ${\vec{v}}_{n}$ is pre- or post-multiplied by any rotation matrix for all *n*, the transformed vector ${\vec{w}}_{n}$ remains unchanged. Note that for this transformation to be orientation invariant, the measured sequence ${\vec{v}}_{n}$ needs to be multiplied by the *same* rotation matrix for all *n*; that is, the sensor can be placed at any orientation at some given position on the body, but its orientation with respect to the body must remain the same during the short time period over which data are processed. This is a necessary restriction because we preserve the *change* in the orientation of measurement vectors ${\vec{v}}_{n}$ in the transformation over time, which provides information about the orientation change of the body if the sensor rotates with the body rather than rotating freely.

To prove the orientation invariance of the transformation $T_{\mathrm{heuristic}}$ mathematically, assume that the sensor is placed at a different orientation and the acquired data are ${\vec{v}}_{n}{\;}^{\prime }=R{\vec{v}}_{n}\;\;\;\;\forall n$, where R is a rotation matrix that is constant over *n*. Then, we need to prove that its transformation ${\vec{w}}_{n}{\;}^{\prime }$ is the same as ${\vec{w}}_{n}$:

(3) $${\vec{w}}_{n}={\vec{w}}_{n}{\;}^{\prime }\;\;\;\;\forall n\;\mathrm{where}\;{\vec{v}}_{n}\overset{T_{\mathrm{heuristic}}}{\to }{\vec{w}}_{n}\;\mathrm{and}\;{\vec{v}}_{n}{\;}^{\prime }\overset{T_{\mathrm{heuristic}}}{\to }{\vec{w}}_{n}{\;}^{\prime }$$

For the proof, note the following facts: (1) multiplying a vector by a rotation matrix does not change its norm; (2) multiplying two vectors by the same rotation matrix affects neither the angle between them nor their inner product; (For the proof, let $\alpha_{n}=∠\left({\vec{v}}_{n},{\vec{v}}_{n+1}\right)$. Then,

$$∠\left(R{\vec{v}}_{n},R{\vec{v}}_{n+1}\right)=\cos^{-1}\left(\frac{\left(R{\vec{v}}_{n}\right)\cdot \left(R{\vec{v}}_{n+1}\right)}{∥R{\vec{v}}_{n}∥∥R{\vec{v}}_{n+1}∥}\right)=\cos^{-1}\left(\frac{{\vec{v}}_{n}\cdot {\vec{v}}_{n+1}}{∥{\vec{v}}_{n}∥∥{\vec{v}}_{n+1}∥}\right)=\alpha_{n}\;\;\;\;\;\mathrm{for}\;\mathrm{any}\;\mathrm{rotation}\;\mathrm{matrix}\;R.)$$

and (3) if a time-varying vector is multiplied by a constant rotation matrix over time, its first- and second-order differences are also multiplied by the same rotation matrix. (For the proof, let $\Delta {\vec{v}}_{n}={\vec{v}}_{n+1}-{\vec{v}}_{n}$ and $\;\Delta \;\;\;\;\;\;\Delta \;{\vec{v}}_{n}=\Delta {\vec{v}}_{n+1}-\Delta {\vec{v}}_{n}$. Then,

$$\begin{array}{ccc} \Delta \;\left(R{\vec{v}}_{n}\right) & = & R{\vec{v}}_{n+1}-R{\vec{v}}_{n}=R\;\Delta {\vec{v}}_{n}\\ \mathrm{and}\;\;\;\Delta \;\;\;\;\;\;\Delta \;\;\left(R{\vec{v}}_{n}\right) & = & \Delta \;\left(R{\vec{v}}_{n+1}\right)-\Delta \;\left(R{\vec{v}}_{n}\right)=R\Delta {\vec{v}}_{n+1}-R\Delta {\vec{v}}_{n}=R\;\;\Delta \;\;\;\;\;\;\Delta \;{\vec{v}}_{n}\;\;\;\;\;\mathrm{for}\;\mathrm{any}\;\mathrm{rotation}\;\mathrm{matrix}\;R.) \end{array}$$

Using these facts, we prove Equation (3) for the first six dimensions of the heuristic OIT:

(4a) $$\begin{array}{cc} w_{1}^{\prime }\left[n\right] & =∥R{\vec{v}}_{n}∥=∥{\vec{v}}_{n}∥=w_{1}\left[n\right] \end{array}$$

(4b) $$\begin{array}{cc} w_{2}^{\prime }\left[n\right] & =∥\Delta \;\left(R{\vec{v}}_{n}\right)∥=∥R\;\Delta {\vec{v}}_{n}∥=∥\Delta {\vec{v}}_{n}∥=w_{2}\left[n\right] \end{array}$$

(4c) $$\begin{array}{cc} w_{3}^{\prime }\left[n\right] & =∥\;\Delta \;\;\;\;\;\;\Delta \;\;\left(R{\vec{v}}_{n}\right)∥=∥R\;\;\Delta \;\;\;\;\;\;\Delta \;{\vec{v}}_{n}∥=∥\;\Delta \;\;\;\;\;\;\Delta \;{\vec{v}}_{n}∥=w_{3}\left[n\right] \end{array}$$

(4d) $$\begin{array}{cc} w_{4}^{\prime }\left[n\right] & =∠\left(R{\vec{v}}_{n},R{\vec{v}}_{n+1}\right)=∠\left({\vec{v}}_{n},{\vec{v}}_{n+1}\right)=w_{4}\left[n\right] \end{array}$$

(4e) $$\begin{array}{cc} w_{5}^{\prime }\left[n\right] & =∠\left(\Delta \;\left(R{\vec{v}}_{n}\right),\Delta \;\left(R{\vec{v}}_{n+1}\right)\right)=∠\left(R\;\Delta {\vec{v}}_{n},R\;\Delta {\vec{v}}_{n+1}\right)=∠\left(\Delta {\vec{v}}_{n},\Delta {\vec{v}}_{n+1}\right)=w_{5}\left[n\right] \end{array}$$

(4f) $$\begin{array}{cc} w_{6}^{\prime }\left[n\right] & =∠\left(\;\Delta \;\;\;\;\;\;\Delta \;\;\left(R{\vec{v}}_{n}\right),\;\Delta \;\;\;\;\;\;\Delta \;\;\left(R{\vec{v}}_{n+1}\right)\right)=∠\left(R\;\;\Delta \;\;\;\;\;\;\Delta \;{\vec{v}}_{n},R\;\;\Delta \;\;\;\;\;\;\Delta \;{\vec{v}}_{n+1}\right)=∠\left(\;\Delta \;\;\;\;\;\;\Delta \;{\vec{v}}_{n},\;\Delta \;\;\;\;\;\;\Delta \;{\vec{v}}_{n+1}\right)=w_{6}\left[n\right] \end{array}$$

For the remaining axes, note that if any two vectors are multiplied by the same rotation matrix, the rotation axis between them also rotates in the same way. To prove this, let ${\vec{p}}_{n}{\;}^{\prime }={\vec{v}}_{n}{\;}^{\prime }\times {\vec{v}}_{n+1}\;\;\;\;\;{\;}^{\prime }\;\;\;\;\;$ be the rotation axis between ${\vec{v}}_{n}{\;}^{\prime }$ and ${\vec{v}}_{n+1}\;\;\;\;\;{\;}^{\prime }\;\;\;\;\;$. Then,

(5) $${\vec{p}}_{n}{\;}^{\prime }={\vec{v}}_{n}{\;}^{\prime }\times {\vec{v}}_{n+1}\;\;\;\;\;{\;}^{\prime }\;\;\;\;\;=\left(R{\vec{v}}_{n}\right)\times \left(R{\vec{v}}_{n+1}\right)=R\left({\vec{v}}_{n}\times {\vec{v}}_{n+1}\right)=R{\vec{p}}_{n}$$

The rotation axes ${\vec{q}}_{n}$ and ${\vec{r}}_{n}$ also rotate in the same way as ${\vec{v}}_{n}$ rotates. Based on these observations, we prove Equation (3) for the remaining dimensions:

(6a) $$\begin{array}{ccccc} w_{7}^{\prime }\left[n\right] & =∠\left({\vec{p}}_{n}{\;}^{\prime },{\vec{p}}_{n+1}\;\;\;\;\;{\;}^{\prime }\;\;\;\;\;\right) & =∠\left(R{\vec{p}}_{n},R{\vec{p}}_{n+1}\right) & =∠\left({\vec{p}}_{n},{\vec{p}}_{n+1}\right) & =w_{7}\left[n\right] \end{array}$$

(6b) $$\begin{array}{ccccc} w_{8}^{\prime }\left[n\right] & =∠\left({\vec{q}}_{n}{\;}^{\prime },{\vec{q}}_{n+1}\;\;\;\;\;{\;}^{\prime }\;\;\;\;\;\right) & =∠\left(R{\vec{q}}_{n},R{\vec{q}}_{n+1}\right) & =∠\left({\vec{q}}_{n},{\vec{q}}_{n+1}\right) & =w_{8}\left[n\right] \end{array}$$

(6c) $$\begin{array}{ccccc} w_{9}^{\prime }\left[n\right] & =∠\left({\vec{r}}_{n}{\;}^{\prime },{\vec{r}}_{n+1}\;\;\;\;\;{\;}^{\prime }\;\;\;\;\;\right) & =∠\left(R{\vec{r}}_{n},R{\vec{r}}_{n+1}\right) & =∠\left({\vec{r}}_{n},{\vec{r}}_{n+1}\right) & =w_{9}\left[n\right] \end{array}$$

Therefore, the orientation invariance of the heuristic OIT is proven.

### 3.2. Orientation-Invariant Transformation Based on Singular Value Decomposition

As an alternative to the heuristic approach, orientation invariance can be achieved by singular value decomposition [28]. In the SVD approach, the x,y,z axes of the original tri-axial sensor are transformed to three principal axes that are orthogonal to each other and along which the variance of the data is the largest. The directions of the principal axes, hence the transformation, depends on the data to be transformed. The motivation for using SVD to achieve orientation invariance is that when the data constellation is rotated as a whole, the principal axes also rotate in the same way, and the representation of the data in terms of the principal axes remains the same.

To apply SVD, data acquired from each tri-axial sensor are represented as a matrix V of size 3×N, with the rows corresponding to the x,y,z axes and the columns representing the time samples:

(7) $$V=\left[\begin{array}{cccc} {\vec{v}}_{0} & {\vec{v}}_{1} & \cdots & {\vec{v}}_{N} \end{array}\right]$$

Then, V is decomposed into three matrices by SVD as

(8) $$V=U\Sigma W^{T}$$

In general, for complex V, U is a 3×3 unitary matrix, Σ is a 3×N rectangular diagonal matrix containing the singular values along the diagonal, and W is an N×N unitary matrix. In our application, V is real, so U and W are real unitary, hence, orthonormal matrices that satisfy $U^{T}U=UU^{T}=I_{3\times 3}$ and $W^{T}W=WW^{T}=I_{N\times N}$, where I is the identity matrix. The matrix U can also be viewed as a 3×3 rotation matrix.

Since the matrix V only has three rows, its rank is at most three, and only the first three singular values can be non-zero. Hence, SVD can be represented more compactly by considering only the first three columns of Σ and W, in which case their sizes become 3×3 and N×3, respectively. This compact representation will be used in the rest of the article, where W is no longer unitary because it is not square, but has orthonormal columns that satisfy $W^{T}W=I_{3\times 3}$.

Changing the orientation of a sensor unit is equivalent to rotating the measurement vectors for each time sample in the same way; that is, pre-multiplying V by a rotation matrix R:

(9) $$\tilde{V}=RV$$

V is constant over time because it is assumed that the sensor orientation with respect to the body part onto which the sensor is placed remains the same while acquiring the data stored in V, as done in the heuristic OIT. The SVD of the rotated data matrix $\tilde{V}$ becomes

(10) $$\tilde{V}=R\left(U\Sigma W^{T}\right)=\left(RU\right)\Sigma W^{T}=\tilde{U}\Sigma W^{T}$$

where $\tilde{U}=RU$ because the product of two rotation matrices is another rotation matrix, and the SVD representation is *almost unique* [29] up to the signs of the columns of U and W. In other words, if a principal vector ${\vec{u}}_{i}$ (the *i*th column of U, where i=1,2,3) is selected in the opposite direction, the variance along that axis is still maximized and the decomposition can be preserved by negating the corresponding column of W. (Another ambiguity in SVD is that the principal vectors can be selected in any direction in case of degenerateness, that is, when V is not full-rank. This situation is not observed in experimental data because of the presence of noise.)

Because of the almost-uniqueness property of SVD, the matrices Σ and W are not affected by the sensor orientation (up to the signs of the columns of W). Therefore, the proposed SVD-based OIT omits the leftmost matrix and takes $\Sigma W^{T}$ as the part of the data that is invariant to sensor orientation (up to the signs of the resulting axes). Then, the SVD-based OIT can be represented as

(11) $$T_{\mathrm{SVD}}:V\to \Sigma W^{T}$$

This transformation is equivalent to a rotational transformation because

(12) $$\Sigma W^{T}=\left(U^{T}U\right)\Sigma W^{T}=U^{T}\left(U\Sigma W^{T}\right)=U^{T}V$$

and $U^{T}$ is the corresponding rotation matrix. Note that the rotation may be right- or left-handed, that is, proper or improper because detU=±1.

The SVD-based OIT rotates the measurement vectors in 3-D space such that the variance of the data along the first principal axis ${\vec{u}}_{1}$ is the largest, followed by the second principal axis ${\vec{u}}_{2}$, which is orthogonal to ${\vec{u}}_{1}$, and followed by the third axis ${\vec{u}}_{3}$, which is orthogonal to both ${\vec{u}}_{1}$ and ${\vec{u}}_{2}$. Thus, if all the vectors are rotated in the same way because of a different sensor orientation, the rotation $U^{T}$ will change accordingly to yield the same transformed sequence (up to the signs of the axes). Mathematically, if the data matrix is rotated as in Equation (9), the same transformed data, $\Sigma W^{T}$, must be obtained (except for the signs of the rows). Hence, using the fact that RU is also a rotation matrix composed of two rotations, one can write

(13) $$\Sigma W^{T}=\left[{\left(\mathrm{RU}\right)}^{T}\left(\mathrm{RU}\right)\right]\Sigma W^{T}={\left(\mathrm{RU}\right)}^{T}\left[\left(\mathrm{RU}\right)\Sigma W^{T}\right]={\left(\mathrm{RU}\right)}^{T}\tilde{V}$$

which reveals that the new rotation matrix of the transformation is ${\left(\mathrm{RU}\right)}^{T}$.

**Figure 2:**
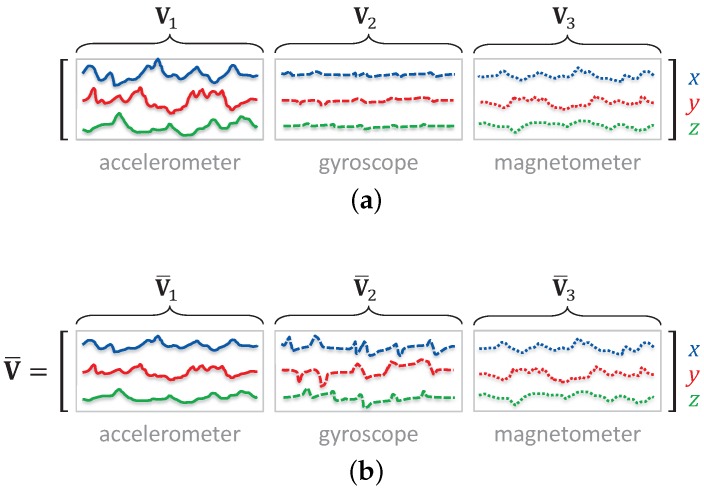
Concatenation of the sequences of different sensor types. (**a**) Accelerometer, gyroscope, and magnetometer sequences are concatenated along the time-sample dimension to obtain a joint 3×3 transformation; and (**b**) the three sequences are normalized to have unit variance (over the whole dataset) before applying SVD-based OIT.

If the unit contains more than one type of sensor (e.g., an accelerometer and a gyroscope), all the sensors have the same orientation with respect to the body part the sensor unit is placed on, ignoring the misalignment errors between the sensors in the same unit [30]. In this case, the same rotational transformation should be applied to the data acquired by all the sensors in the same unit. Let $V_{1},V_{2},\;\;\ldots ,V_{S}$ be the data matrices of sensors 1–*S*, defined as in Equation (7). These are concatenated as $\left[\begin{array}{cccc} V_{1} & V_{2} & \cdots & V_{S} \end{array}\right]$ to obtain a joint transformation, as illustrated in Figure 2a for the first dataset (dataset A). In the figure, sequences of the three sensor types, namely the accelerometer, gyroscope, and magnetometer, are concatenated along the time-sample dimension. Gyroscope sequences have the smallest variance and accelerometer sequences have the largest. However, the more the data of a sensor vary, the more the SVD transformation is affected, and that sensor will have a greater contribution. Hence, we normalize the data of the different sensor types to equalize their effect on the transformation: In each dataset, we scale the sequences of each sensor type to have unit variance over the whole dataset. Then, we concatenate the normalized sequences (indicated by an overbar) as $\bar{V}=\left[\begin{array}{cccc} {\bar{V}}_{1} & {\bar{V}}_{2} & \cdots & {\bar{V}}_{S} \end{array}\right]$ and use it in place of V in Equations (8)–(12). The normalized sequences are shown in Figure 2b. Finally, we apply the SVD-based OIT, where a single 3×3 rotational transformation is employed for the same segment of all the sensor sequences acquired from the same sensor unit.

**Figure 3:**
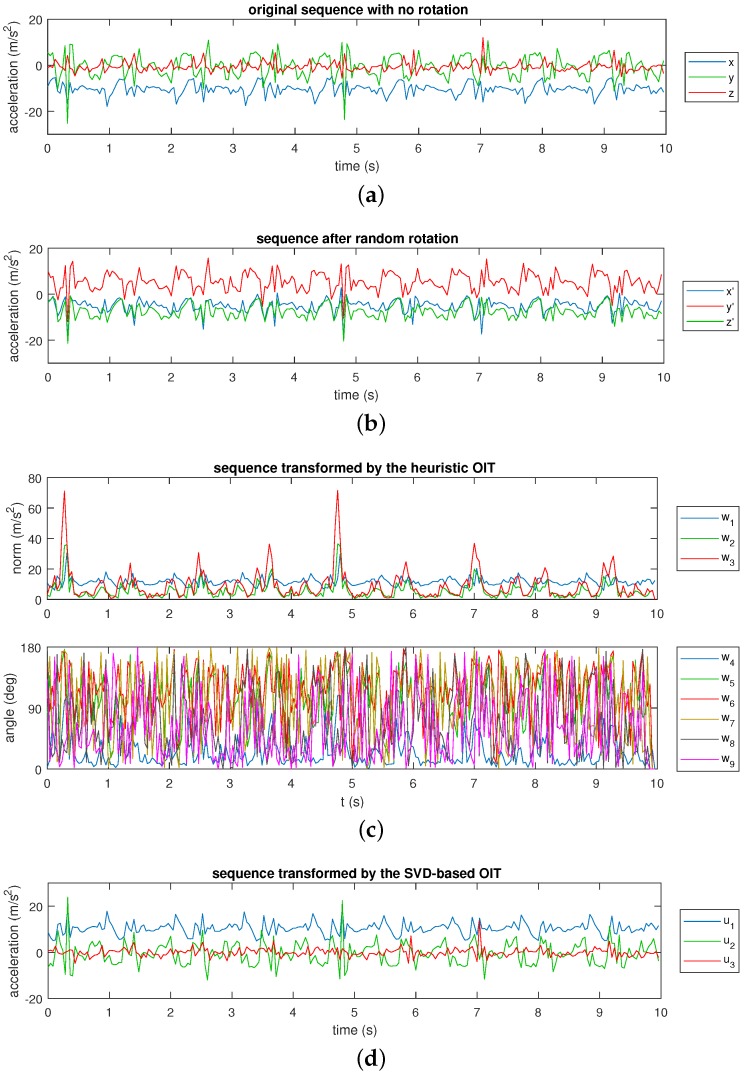
The original, randomly rotated and orientation-invariant sensor sequences. (**a**) Original and (**b**) randomly rotated accelerometer sequences while performing A${}_{10}$ in dataset A. Orientation-invariant sequences transformed by the (**c**) heuristic and (**d**) SVD-based OIT.

As an example, the 3-D sequence of the accelerometer on the left leg of the first subject as he performs the tenth activity (A${}_{10}$) in our activity dataset (dataset A in Section 4.1) is plotted in Figure 3a. The sequence is rotated arbitrarily in 3-D space and plotted in Figure 3b. For this specific example, the rotation angle sequence is $-23.8^{\circ },-54.3^{\circ },12.9^{\circ }$ about the z,y, and *x* axes, respectively. To obtain orientation-invariant sequences, the original sequence (or, equivalently, the rotated sequence) is transformed by the heuristic OIT (Figure 3c) and the SVD-based OIT (Figure 3d). Note that the sequences in Figure 3c,d can be obtained by transforming either the original sequence in Figure 3a or its rotated form in Figure 3b, or by any other arbitrarily rotated form of Figure 3a. It is observed that the quasi-periodic nature of the data is preserved in both transformations. Since we observe in Figure 3c that the last two elements of the sequence transformed by the heuristic OIT contains much noise, we did not consider including differences of the sensor sequences beyond second order.

## 4. Experimental Methodology and Results

### 4.1. Datasets

**Figure 4:**
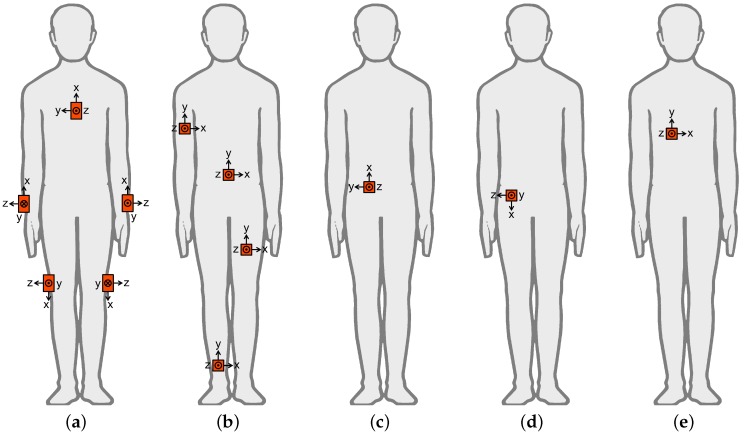
(**a**–**e**) Sensor configurations in datasets A–E. The body drawing in the figure is from http://www.clker.com/clipart-male-figure-outline.html onto which sensor units were added by the authors.

We use five publicly available datasets recorded by different research groups to observe the effects of the proposed transformations on the acquired data. The datasets are labeled A–E and their attributes are provided in Table 1. The sensor configurations for the datasets are shown in Figure 4.

Dataset A was acquired by our research group [11,35,36] using five Xsens MTx wearable sensor units containing tri-axial accelerometers, gyroscopes, and magnetometers [37]. Nineteen activities were considered, including random activities such as playing basketball (see Table 1 for the list of activities in the datasets). Among the five datasets, A is the largest, including a wide range of activities and employing a small network of five sensor units. Unlike in the other four datasets, in dataset A, each subject performs each activity for an equal amount of time. Dataset B utilizes four accelerometers and considers five basic activities, some of which are transitional activities, such as sitting down [31,38]. However, this property is not used in the classification process. Dataset C considers six basic activities and utilizes a smartphone containing an accelerometer and a gyroscope [32,39]. Using a high-pass filter, the gravitational component of the total acceleration is removed and an additional 3-D sequence is obtained. This dataset has the largest number of subjects among the five datasets. Dataset D includes 12 activities and utilizes a single sensor unit containing an accelerometer and a gyroscope [33]. Unlike in the other four datasets, the subjects have a diverse range of age, height, and weight attributes. Dataset E utilizes a single tri-axial accelerometer placed on the chest [34,40]. Most of the 15 subjects are male. Seven activities are considered, some of which are compound activities that contain more than one activity; for example, one of the activities comprises standing up, walking, and going up/down stairs. Some activity pairs seem to be difficult to distinguish, such as “walking” versus “walking and talking with someone.” This dataset contains the smallest number of features per segment because only a single tri-axial sensor is used.

### 4.2. Activity Recognition

**Figure 5:**
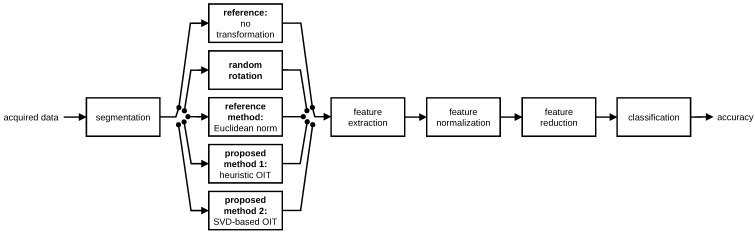
Activity recognition paradigm.

In activity recognition, a procedure similar to that in [35,36] is followed, whose block diagram is provided in Figure 5. In the pre-processing stage, the following steps are taken in order: the data sequences are segmented into time windows of fixed duration, one of the two OIT methods is applied if orientation invariance is desired, features are extracted from each segment, features are normalized, and PCA is applied to reduce the number of features. Then, classification is performed with four different classifiers and their accuracy is calculated using two cross-validation techniques.

#### 4.2.1. Pre-Processing

First, the recorded data sequences are divided into non-overlapping segments of five seconds’ duration each for datasets A, B, D, and E. Dataset C is originally divided into 50% overlapping segments of 2.56 s duration each and the original segments are used for this dataset. For all datasets, each segment belongs to a continuous recording of a single activity performed by one of the subjects. The number of segments extracted from datasets A–E are 9120, 4130, 10,299, 5353, and 7345, respectively.

Following segmentation, one of the two proposed OIT techniques is applied to each segment of the data if orientation invariance is desired. Five cases are considered to observe the effects of sensor rotation on the classification process and to observe the improvement obtained with the proposed transformations:

- **Reference case** is the standard (ordinary) activity recognition scheme with fixed sensor positions and orientations. In this case, originally recorded sequences are used without applying any transformation.
- **Random rotation case** simulates the situation where each sensor unit is placed at a fixed position at any orientation. We use the original dataset by synthetically rotating the data to make a fair comparison between reference and random rotation cases. Tri-axial recordings of each sensor unit in each segment are randomly rotated in 3-D space to observe the performance of the system when the units are placed at random orientations. To this end, for each segment of each unit of a given dataset, we generate a random rotation matrix R and pre-multiply each of the three-element measurement vectors belonging to that segment (for the accelerometer, gyroscope, and magnetometer if available) by this rotation matrix as $\tilde{V}=RV$. The rotation matrix is calculated from yaw, pitch, and roll angles θ,ϕ,ψ that are randomly generated in the interval [0,2π) radians:
  (14) $$R=\left[\begin{array}{ccc} 1 & 0 & 0\\ 0 & \cos\theta & -\sin\theta \\ 0 & \sin\theta & \cos\theta \end{array}\right]\left[\begin{array}{ccc} \cos\phi & 0 & \sin\phi \\ 0 & 1 & 0\\ -\sin\phi & 0 & \cos\phi \end{array}\right]\left[\begin{array}{ccc} \cos\psi & -\sin\psi & 0\\ \sin\psi & \cos\psi & 0\\ 0 & 0 & 1 \end{array}\right]$$
  Note that while all of the sensor types in the same unit are rotated in the same way for a given segment, each segment recorded from each sensor unit for each dataset is rotated differently (by a different random rotation matrix).
- **Euclidean norm method** takes the Euclidean norm of each 3-D sensor sequence at each time sample, and uses only the norms (as functions of the time sample) in classification. This is indeed a basic but proper OIT technique, which corresponds to the first dimension of the transformed signal, $w_{1}\left[n\right]$, in the heuristic OIT. It has been used in some studies to obtain a scalar quantity as a feature [41], to achieve orientation invariance in the simplest possible way [17,42], or to incorporate additional information such as the energy expenditure estimate of the subject [43]. Taking the Euclidean norm reduces the number of axes by a factor of three.
- **Proposed method 1** corresponds to the heuristic OIT technique. The time-domain sequence contained in each segment of each tri-axial sensor in each unit is transformed to yield a 9-D orientation-invariant time-domain sequence. As a consequence, dimensionality of the time-domain data increases by a factor of three (from three to nine). We also consider taking only the first three or the first six elements of the transformation. (Throughout this article, all of the nine elements of the heuristic OIT are considered unless stated otherwise.)
- **Proposed method 2** corresponds to the SVD-based OIT. A single transformation is calculated for all the sensor types in each sensor unit, again independently for each time segment, as explained in Section 3.2. The dimensionality is not affected by this transformation, unlike the Euclidean norm method and proposed method 1.

Although the sensor units are placed on the body at the same orientation during data acquisition, the applied transformations in the last three cases remove the orientation information from the data, simulating the case where each sensor unit is placed at any orientation on the body at a fixed position. Thus, a fair comparison can be made among the five cases based on the same experimental data.

For each segment, statistical features are extracted from each axis of the (possibly transformed) data and are concatenated to construct the feature vector associated with that segment. For instance, for dataset A and the reference case, there are 5units×9sensors=45axes in total, and the features are extracted separately from each of these 45 axes over the given time segment, and concatenated into a single feature vector associated with that particular segment. The following features are considered: mean, variance, skewness, kurtosis, certain coefficients of the autocorrelation sequence (Every fifth autocorrelation sample up to the 50th is used. The variance is included once as the first autocorrelation sample. Fewer coefficients may be used depending on the length of the segment), and the five largest discrete Fourier transform (DFT) peaks with the corresponding frequencies. (The separation between any two peaks in the DFT sequence is taken to be at least 11 samples. A smaller number of peaks can be used depending on the segment duration.) The number of features are 1170, 276, 234, 156, and 78 for datasets A–E, respectively, for the reference case. Following feature extraction, the features are normalized to the interval [0,1] for each subject in each dataset.

**Figure 6:**
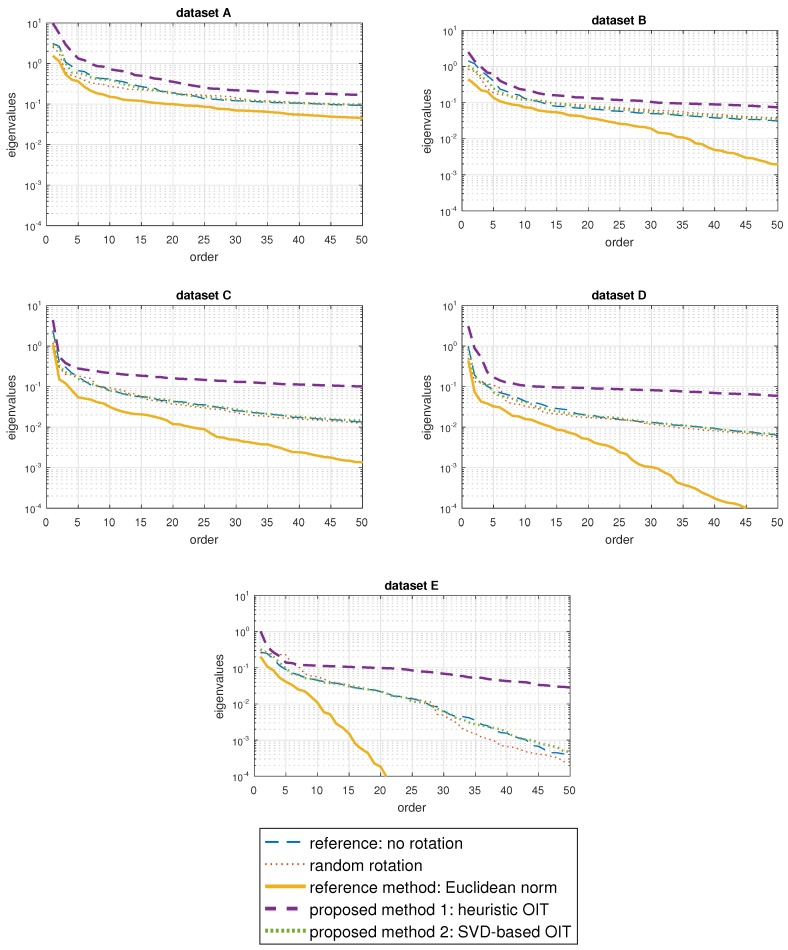
The first 50 eigenvalues of the covariance matrix in descending order for the features extracted from the data transformed according to the five cases.

As the last step of the pre-processing stage, the number of features is reduced through PCA, which linearly transforms the feature space such that the transformed features are sorted in descending order of variance. This approach allows us to consider only the first *M* dimensions in the classification process, decreasing the computational complexity and possibly improving classification if an appropriate value of *M* is chosen. Moreover, it enables us to make a comparison between the different datasets by equalizing the dimensionality of the feature space among them. To select an appropriate value for *M*, the eigenvalues of the covariance matrix of the feature vectors extracted from each of the five cases are sorted in descending order and plotted in Figure 6 for each dataset. M=30 appears to be a suitable choice because there is a dramatic decrease from the first eigenvalue to the 30th in all five datasets.

#### 4.2.2. Classification and Cross Validation

Following feature reduction, classification is performed with four state-of-the-art classifiers. The parameters of the second and the third classifiers are jointly optimized by a grid search for all five cases, the two cross-validation techniques, and the five datasets. The classifiers and the parameter optimization process are explained below.

1. **Bayesian Decision Making (BDM):** To train a BDM classifier, for each activity class a multi-variate Gaussian distribution is fitted using the training feature vectors of that class by using maximum likelihood estimation. This process involves estimating the mean vector and the covariance matrix for each class. Then, for a given test vector, its conditional probability, conditioned on the class information (i.e., the probability given that it belongs to a particular class) can be calculated. The class that maximizes this probability is selected according to the maximum a posteriori (MAP) decision rule [44,45].
2. ***k*-Nearest-Neighbor (*k*-NN):** The *k*-NN classifier requires storing training vectors. A test vector is classified by using majority voting on the classes of the *k* nearest training vectors to the test vector in terms of the Euclidean distance, where *k* is a parameter that takes integer values [44,45]. In this study, *k* values ranging from 1 to 30 have been considered for all cases, cross-validation techniques, and datasets. The value k=7 is found to be suitable and is used throughout this work.
3. **Support Vector Machines (SVM):** The SVM is a binary classifier in which the feature space is separated into two classes by an optimal hyperplane that has the maximum margin [45]. In case the original feature space may not be linearly separable, it can be implicitly and nonlinearly mapped to a higher-dimensional space by using a kernel function, which represents a measure of similarity between two data vectors x and y. There are two commonly used kernels: the Gaussian radial basis function (RBF), $f_{\mathrm{RBF}}\left(x,y\right)=e^{{-\gamma ∥x-y∥}^{2}}$, and the linear kernel, $f_{\mathrm{linear}}\left(x,y\right)=x^{T}y$. In this study, we use the former, which is equivalent to mapping the feature space to a Hilbert space of infinite dimensionality. The reason for this choice is that there is no need to consider the linear kernel if the RBF kernel is used with optimized parameters [46], which is the case here. Then, binary classification is performed according to which side of the hyperplane the test vector resides on. To use the SVM with more than two classes, a one-versus-one approach is followed where a binary SVM classifier is trained for each class pair. A test vector is classified with all pairs of classifiers and the classifier with the highest confidence makes the class decision [47]. The MATLAB toolbox LibSVM is used for the implementation [48]. The two parameters of the SVM classifier, *C* and γ, are optimized jointly over all five cases, both cross-validation techniques, and all five datasets. The parameter *C* is the penalty parameter of the optimization problem of the SVM classifier (see Equation (1) in [49]) and γ is the parameter of the Gaussian RBF kernel described above. A two-level grid search is used to determine the parameter pair that performs the best over all cases, cross-validation techniques, and datasets. In the coarse grid, the parameters are selected as $\left(C,\;\gamma \right)\in \left\{10^{-5},\;10^{-3},\;10^{-1},\;\cdots ,\;10^{15}\right\}\times \left\{10^{-15},\;10^{-13},\;10^{-11},\;\cdots ,\;10^{3}\right\}$ and the best parameter pair is found to be $\left(C^{*},\;\gamma^{*}\right)=\left(10^{1},\;10^{-1}\right)$. Then, a finer grid around $\left(C^{*},\;\gamma^{*}\right)$ on the set $\left(C,\;\gamma \right)\in 100\;P\times P$, with $P=\left\{0.01,\;0.05,\;0.1,\;0.2,\;0.3,\;0.4,\;0.5,\;0.7,\;1,\;3,\;5\right\}$ reveals the best parameter pair $\left(C^{**},\;\gamma^{**}\right)=\left(40,\;0.2\right)$, which is used in all five cases, cross-validation techniques, and datasets considered in this study.
4. **Artificial Neural Networks (ANN):** An ANN consists of neurons, each of which produces an output that is a nonlinear function (called the activation function) of a weighted linear combination of multiple inputs and a constant. In this study, the sigmoid function, $g\left(x\right)={\left(1+e^{-x}\right)}^{-1}$, is used as the activation function [45]. A multi-layer ANN consists of several layers of neurons. The inputs to the first layer are the elements of the feature vector. In the last layer, a neuron is allocated to each of the *K* classes. The number of hidden-layer neurons is selected as the nearest integer to $\frac{1}{2}\left(\frac{\ln\left(2K\right)}{\ln2}+2K-1\right)$, depending on the number of classes *K*. (As a rule of thumb, each class is assumed to have two linearly separable subclasses. Then, the number of neurons in the hidden layer is selected as the average of the optimistic and pessimistic cases. In the former, $\frac{\ln\left(2K\right)}{\ln2}$ neurons are required to have the hyperplanes intersect at different positions, whereas in the latter, 2K−1 neurons are required for parallel hyperplanes [50].) Training an ANN can be implemented in various ways and determines the weights of the linear combination for each neuron. The desired output is one for the neuron corresponding to the class of the input vector and zero for the output neurons of the other classes. The back-propagation algorithm is used for training, which iteratively minimizes the errors in the neuron outputs in the least-squares sense, starting from the last layer and proceeding backwards [51]. The weights are initialized with a uniform random distribution in [0,0.2] and the learning rate is chosen as 0.3. An adaptive stopping criterion is used, which terminates the algorithm at the *i*th epoch (that is, when each training vector has been used exactly *i* times) if $\min\left\{E_{i-9},\;E_{i-8},\;\cdots ,\;E_{i}\right\}>E_{i-10}-0.01$, where $E_{i}$ is the average of the sum of the squared errors over all the training vectors in the last layer’s outputs at the *i*th epoch. In other words, the algorithm stops when the errors at (any of) the last 10 epochs are not significantly smaller than the error at the 11th epoch from the end. In classification, a test vector is given as the input to the ANN and the output neuron with the maximum output indicates the class decision.

The accuracies of the classifiers are determined by two cross-validation techniques: *P*-fold and leave-one-subject-out (L1O). In *P*-fold cross validation, the dataset is randomly divided into P=10 equal partitions and the data in each partition are classified with a classifier trained by the data in all the remaining partitions. L1O is the same as *P* fold except that partitioning is done based on the subjects so that each partition contains the data of a particular subject. L1O is more challenging than *P* fold for the classifiers because in the former, the training and test sets contain different subjects’ data.

### 4.3. Results

We naturally expect the accuracy achieved with the proposed transformations to be lower compared to the reference case because neither of the two transformations preserves the direction of the gravity vector detected by the accelerometers nor the direction of the Earth’s magnetic North measured by the magnetometers. After transforming, absolute sensor orientations are no longer available. Removing this information is necessary to provide the user the flexibility to place the sensor units at any orientation.

**Figure 7:**
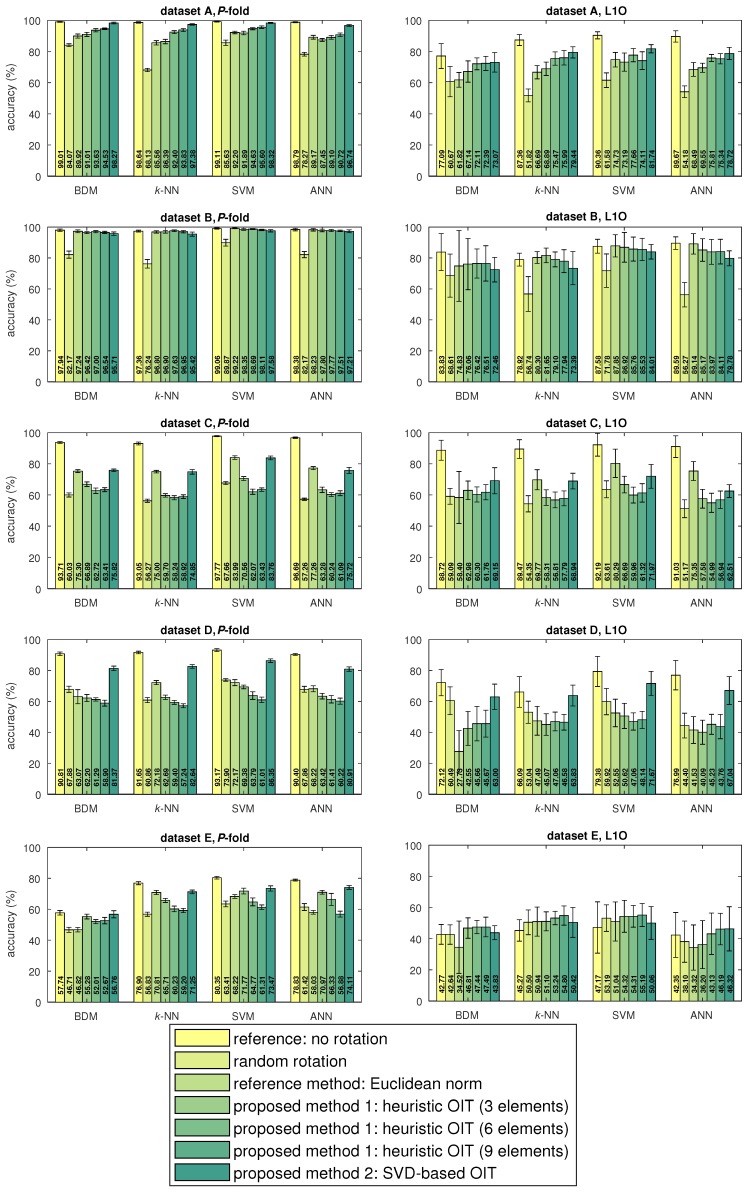
Accuracies shown as bars or horizontal lines for all the cases, datasets, classifiers, and cross-validation techniques. The vertical sticks indicate plus/minus two standard deviations around the mean over the cross-validation iterations.

The activity recognition accuracies for datasets A–E are shown in Figure 7, along with the standard deviations over the cross-validation iterations. For each dataset, the classifier accuracies are presented for the five cases for each cross-validation technique. We observe that when the standard activity recognition system is used with randomly oriented sensors (the random rotation case), the accuracy drops by 21.21% on the average, compared to the reference case. Using only the Euclidean norm improves the accuracy drop for datasets A–C, and causes an average degradation of 13.50% in accuracy compared to the reference case, over all datasets. We also observe that both of the proposed OIT techniques significantly improve the accuracy drop compared to the random rotation case in most situations. On the average, proposed methods 1 (with 9 elements) and 2 decrease the accuracy by 15.54% and 7.56%, respectively, compared to the reference case; hence, the latter is superior to the former most of the time. When the first three or the first six elements of the heuristic OIT are used, the performance depends on the dataset and the cross-validation technique used and is comparable to using all nine elements. The accuracy obtained by using the SVD-based OIT is comparable with the reference case for all datasets except for C for which it is lower.

The most accurate classifier, in general, is the SVM; its accuracy is especially greater than the other classifiers when the sensors are oriented randomly. This result shows that the SVM is robust against challenges associated with the classification problem and imperfections in the data, even though the same parameter values are used for the SVM classifier throughout the study. The robustness of the SVM in different problems is consistent with the results obtained in [17]. The second most robust classifier is BDM, which is also more accurate than most of the other classifiers for random rotation for all datasets. We attribute the robustness of BDM to its ”coarseness” in classification, which improves the accuracy in classifying imperfect data. In other words, because each segment in the training and test data is rotated randomly and differently, the feature vectors are scattered in the feature space. In this case, one needs to train a classifier that will not separate the feature space haphazardly based on individual samples, but rather consider the simple common properties of the feature vector constellations of the classes. Binary decision making realizes this successfully, fitting a smooth Gaussian distribution to the training data of each class. However, the *k*-NN classifier, for instance, partitions the feature space into regions with complicated boundaries and performs worse for randomly rotated data.

Since we use the same methodology to classify the activities in all datasets, we are able to make a fair comparison between the datasets. Referring to Figure 7, we observe that the activity recognition accuracy highly differs among the datasets even for the reference case where no transformation is applied: Datasets D and E result in lower accuracy than datasets A–C for all four classifiers. In particular, the classifiers perform poorly for dataset E, especially for L1O cross validation, where most of the segments are incorrectly classified. This result shows that a single tri-axial accelerometer worn on the chest is not sufficient to recognize relatively complicated activities, such as working at a computer (E${}_{1}$) or talking while standing (E${}_{7}$). Rotating or transforming the data does not have a significant effect on the results for dataset E and L1O cross validation, indicating that the recorded data do not contain sufficient information about the activities. We also observe in all datasets that the L1O cross-validation technique results in much lower accuracy than *P* fold because of the variations in the data across the subjects who perform the activities [52].

## 5. Discussion

We have not recorded a new dataset for incorrectly oriented sensor units in this study. The first reason for this choice is that it would not have been possible to compare the five cases based on the same dataset because we would not have been able to obtain the results in the reference case using a dataset recorded with different sensor orientations. Considering that there are usually significant variations in the data recorded from activities performed by different subjects and by the same subject at different times [52,53], comparing the five cases based on different datasets would not be fair. The second reason is that the proposed OITs completely remove the absolute orientation information from the data, which means that the transformed sequences would be exactly the same if the sensor units were oriented differently. A third reason is the difficulty of selecting the incorrect sensor orientations considered in the new dataset because this would highly affect the results of random rotation.

In this study, we assume that each sensor unit may be placed at any orientation at a given position but the orientation on the body must remain the same in the short term. We make this assumption because we wish to preserve the information related to the rotational motion of the body related to the activities performed and only remove that related to the absolute orientation of the sensors. To this end, in the heuristic OIT, we extract some quantities from the sensor sequences and their time differences that are invariant to sensor orientation. If the sensor orientation with respect to the body changes over time, these difference sequences will be affected. However, the heuristic OIT uses differences spanning at most four consecutive time samples, which correspond to a duration of three sampling periods (0.12, 0.375, 0.06, 0.03, and 0.06 sec in datasets A–E, respectively). Thus, it is sufficient to maintain the sensor orientations for three sampling periods to obtain uncorrupted transformed sequences. This result translates into practice, where the sensor orientations are allowed to deviate slowly provided that the deviation over three sampling periods is negligible. This property is not valid for the SVD-based OIT, which requires that the sensor orientations with respect to the body remain the same throughout the time period the transformation is applied (one segment). However, since each segment is transformed independently in both the training and test phases, the sensor orientations in each segment may be completely different. This result would have no effect on the transformed sequences nor the accuracy.

Unlike some studies that assume correct sensor placement in the training phase, such as [10], we allow users of wearable systems the flexibility to place the sensors at any orientation during both the training and test phases for both OIT techniques. Many studies consider only a small and finite number of orientations, whereas in our approach, orientation angles can take values over a continuum. This method is advantageous because of the inevitable deviations in sensor placement over time. We also do not make any assumptions regarding the nature of the daily activities. For instance, in [13], to estimate the directions of the forward-backward and vertical axes of the human body, it is assumed that the long-term average of the acceleration provides the direction of the gravity vector, and most of the variations perpendicular to the vertical axis are along the forward-backward direction of the body. Similar assumptions are made in [14]. These assumptions are not valid in applications such as monitoring elderly, disabled, or injured people, and children who are more likely to place the sensors incorrectly because of these users’ limitations, or in evaluating physical therapy or sports exercises, where the subjects’ body movements can be more vigorous and different than those in daily activities. Thus, we believe that the existing techniques are not applicable to the generic activity recognition framework and that the approaches proposed here allow more flexibility.

The most important advantage of our methodology is that the OIT techniques that we propose can be readily used without much effort at the beginning of the typical activity recognition paradigm (consisting of segmentation, feature extraction and reduction, and classification, Figure 5), provided that rule-based heuristic approaches that rely on the meanings of the raw sensor measurements are not used. The SVD-based OIT can be applied to the raw sensor measurements in any kind of system that processes multi-dimensional time-domain sequences. The only requirement to apply the heuristic OIT is that the system should be able to process up to 9-D time-domain sequences instead of 3-D ones.

Runtime Analysis:

To assess the computational cost of pre-processing the sequences, the runtimes of the proposed OIT techniques and the Euclidean norm method are provided in Table 2 for each dataset. We observe that the calculation of the heuristic OIT takes the longest, followed by the SVD-based OIT, and the Euclidean norm approach. As the number of elements included in heuristic OIT is increased from 1 (Euclidean norm) to 3 to 6 to 9, the runtime naturally increases. The 3-element and 6-element versions of the heuristic OIT algorithm could be suitable for deployment on resource-limited platforms for which the calculation of an inverse cosine or a vector dot/cross product is a significant effort.

We also investigate the runtimes of the classifiers that show some variation. The classifiers’ runtimes are presented separately for the five cases and the two cross-validation techniques for dataset A in Table 3. (The processing was performed on 64-bit MATLAB^®^ R2016a running on a laptop with a quad-core processor (Intel^®^ Core^TM^ i7-4720HQ) at 2.6–3.6 GHz and with 16 GB of RAM. For the heuristic OIT, runtimes of the version with nine elements is provided.) In the rows entitled “runtime,” each entry is the sum of the training and classification times of all the test feature vectors in an average cross-validation iteration. It is observed that *k*-NN is significantly faster than the other classifiers, whereas the ANN and SVM are relatively slow. The variation in the runtime across the five cases and the two cross-validation techniques is not as much as the variation across the classifiers.

Each entry in the rows entitled “training time” is the average duration of training a classifier in a *single* *P*-fold or L1O iteration. The ANN and SVM are about three orders of magnitude slower than the other classifiers in training, in exchange for higher accuracy. The *k*-NN classifier does not require any training because it only needs to store the training feature vectors for classification. The training time of BDM does not significantly depend on the data, hence, it is nearly the same for each of the five cases and the two cross-validation techniques. On the other hand, the training times of the SVM and ANN highly differ across the five cases and the two cross-validation techniques, and training is faster in the reference case and proposed method 2.

The rows entitled “classification time” contain the average durations of classifying *a single* test feature vector for each case and each cross-validation technique. In all cases, BDM has the longest classification time, whereas ANN has the shortest. The classification time of the SVM is case dependent, whereas the classification times of the other classifiers are comparable for each of the five cases.

## 6. Conclusions and Future Work

Allowing users to fix wearable sensors at any orientation brings considerable flexibility to wearable systems, especially considering that wearable sensors have diminished in size and become wireless. Furthermore, devices such as smartphones and watches that contain motion sensors are part of many wearable sensing systems, which are prone to be placed at an incorrect orientation. Further, elderly, disabled, and injured people, as well as people with various disorders also use these kind of systems and have a greater tendency to place the sensors incorrectly compared to other users. In prior studies, this fact has often been overlooked and sensors are usually assumed to be fixed correctly on the body. However, it is observed that the existing systems are not robust to incorrectly placed sensors.

Our aim was to solve the generic problem of placing sensors at an incorrect orientation, instead of partially solving both the incorrect position and orientation problems under restrictive assumptions. The results show that both OIT techniques that we propose solve the issue of incorrect sensor orientation in activity recognition, with an average absolute reduction of 11.6% in accuracy. In particular, compared to the reference case, the SVD-based OIT causes an average accuracy degradation of 7.56%, whereas this value is 15.54% for the heuristic OIT. On the other hand, with no transformation, random sensor orientation decreases the accuracy by 21.21% on average, which shows the effectiveness of the transformations that we propose. The use of these transformations requires neither restrictive assumptions about the sensor and activity types nor about the sensor positions. The proposed methodology can be used in the pre-processing stage of existing wearable systems without much effort, making them invariant to sensor orientation.

Even though the focus of the present study is the application of activity recognition, the transformations proposed here can also be exploited in other applications of wearables, such as authentication of users in mobile sensing systems [54], evaluating physical therapy exercises [55], or fall detection and classification [56]. For instance, the study reported in [54] assumes that the motion sensors used for gait-based personal authentication have fixed orientations. In [55], physical therapy exercises are detected and evaluated based on template signals by using time-domain sequences acquired from wearable sensors. Making fall detection and classification algorithms invariant to sensor position and orientation would be another valuable contribution. The proposed OIT techniques can be employed in such applications to allow the sensor units to be placed at any orientation.

Some studies on position invariance, such as [25,26], assume that sensor positions may vary but orientations remain fixed. The next step of our research is to combine the methods proposed here with the methodology presented in those studies to obtain a system invariant to both sensor position and orientation.

## Figures and Tables

**Table 1:** Attributes of the five datasets.

Dataset	A [11]	B [31]	C [32]	D [33]	E [34]
no. of subjects	8	4	30	14	15
no. of activities	19	5	6	12	7
activities	sitting (A${}_{1}$), standing (A${}_{2}$), lying on back and on right side (A${}_{3}$, A${}_{4}$), ascending and descending stairs (A${}_{5}$, A${}_{6}$), standing still in an elevator (A${}_{7}$), moving around in an elevator (A${}_{8}$), walking in a parking lot (A${}_{9}$), walking on a treadmill in flat and $15^{\circ }$ inclined positions at a speed of 4 km/h (A${}_{10}$, A${}_{11}$), running on a treadmill at a speed of 8 km/h (A${}_{12}$), exercising on a stepper (A${}_{13}$), exercising on a cross trainer (A${}_{14}$), cycling on an exercise bike in horizontal and vertical positions (A${}_{15}$, A${}_{16}$), rowing (A${}_{17}$), jumping (A${}_{18}$), and playing basketball (A${}_{19}$)	sitting down (B${}_{1}$), standing up (B${}_{2}$), standing (B${}_{3}$), walking (B${}_{4}$), and sitting (B${}_{5}$)	walking (C${}_{1}$), ascending stairs (C${}_{2}$), descending stairs (C${}_{3}$), sitting (C${}_{4}$), standing (C${}_{5}$), and lying (C${}_{6}$)	walking (D${}_{1}$), walking left and right (D${}_{2}$ and D${}_{3}$), ascending and descending stairs (D${}_{4}$, D${}_{5}$), running forward (D${}_{6}$), jumping (D${}_{7}$), sitting (D${}_{8}$), standing (D${}_{9})$, sleeping (D${}_{10}$), ascending and descending in an elevator (D${}_{11}$, D${}_{12}$)	working at a computer (E${}_{1}$), standing up–walking–ascending/descending stairs (E${}_{2}$), standing (E${}_{3}$), walking (E${}_{4}$), ascending/descending stairs (E${}_{5}$), walking and talking with someone (E${}_{6}$), talking while standing (E${}_{7}$)
no. of non-stationary	15	3	3	9	4
activities	A${}_{5}$–A${}_{19}$	B${}_{1}$, B${}_{2}$, B${}_{4}$	C${}_{1}$–C${}_{3}$	D${}_{1}$–D${}_{7}$, D${}_{11}$, D${}_{12}$	E${}_{2}$, E${}_{4}$–E${}_{6}$
no. of units	5	4	1	1	1
no. of axes per unit	9	3	6	6	3
unit positions	torso	waist	waist	front right hip	chest
	right and left arm	left thigh			
	right and left leg	right ankle			
		right upper arm			
	accelerometer	accelerometer	accelerometer	accelerometer	accelerometer
sensor types	gyroscope		gyroscope	gyroscope	
	magnetometer		(of smartphone)		
dataset duration (h)	13	8	7	7	10
sampling rate (Hz)	25	8	50	100	52
no. of segments	9120	4130	10,299	5353	7345
			(50% overlap)		
segment length (s)	5	5	2.56	5	5
no. of features					
(for the reference case,	1170	276	234	156	78
with no transformation)					

**Table 2:** Runtimes of the three OIT techniques (in sec) for datasets A–E.

Method	Dataset				
	A	B	C	D	E
Euclidean norm	6.597	2.338	5.515	4.123	3.513
proposed method 1: heuristic OIT (3 elements)	28.928	2.226	6.574	5.954	2.763
proposed method 1: heuristic OIT (6 elements)	191.406	10.096	44.059	49.240	21.005
proposed method 1: heuristic OIT (9 elements)	369.243	17.503	84.239	91.445	38.670
proposed method 2: SVD-based OIT	70.034	4.122	20.434	59.737	8.325

**Table 3:** Total runtime (training and classification of all test feature vectors), average training time per single cross-validation iteration, and average classification time per feature vector for dataset A.

		Reference	Euclidean Norm	Random Rotation	Proposed Method 1	Proposed Method 2	Reference	Euclidean Norm	Random Rotation	Proposed Method 1	Proposed Method 2
	**Classifier**	***P*****-Fold**					**L1O**				
**runtime** **(s)**	BDM	1.312	1.617	1.303	1.292	1.309	1.628	1.612	1.688	2.588	2.384
	*k*-NN	0.149	0.156	0.157	0.155	0.153	0.175	0.172	0.185	0.424	0.259
	SVM	13.238	36.050	12.230	30.504	13.645	12.074	28.420	11.700	34.525	17.495
	ANN	8.754	12.796	13.850	14.482	10.118	7.992	9.326	9.131	11.353	9.326
**training** **time** **(s)**	BDM	0.009	0.010	0.008	0.009	0.009	0.009	0.009	0.009	0.014	0.013
	*k*-NN	0.000	0.000	0.000	0.000	0.000	0.000	0.000	0.000	0.000	0.000
	SVM	12.839	30.186	11.681	29.891	13.219	11.636	27.521	11.056	33.580	16.811
	ANN	8.561	12.773	13.826	14.448	10.095	7.966	9.299	9.104	11.313	9.299
**classification** **time** **(ms)**	BDM	1.424	1.757	1.416	1.403	1.421	1.417	1.404	1.469	2.253	2.075
	*k*-NN	0.159	0.166	0.168	0.166	0.163	0.150	0.147	0.159	0.367	0.222
	SVM	0.307	0.722	0.478	0.522	0.342	0.285	0.690	0.463	0.659	0.451
	ANN	0.019	0.018	0.020	0.026	0.017	0.017	0.017	0.017	0.025	0.018

## Abbreviations

The following abbreviations are used in this manuscript:

OIT	Orientation-Invariant Transformation
SVD	Singular Value Decomposition
BDM	Bayesian Decision Making
*k*-NN	*k*-Nearest Neighbor
SVM	Support Vector Machines
ANN	Artificial Neural Networks
PCA	Principal Component Analysis
DFT	Discrete Fourier Transform
RBF	Radial Basis Function
MAP	maximum a posteriori
L1O	Leave One Out
